# Developing and evaluating a national Virtual Community of Practice to support family and friend caregivers of persons living with dementia

**DOI:** 10.1002/alz70858_102169

**Published:** 2025-12-25

**Authors:** Marie‐Lee Yous, Ruthie Zhuang, Sharon Kaasalainen, Carrie A McAiney

**Affiliations:** ^1^ McMaster University, Hamilton, ON, Canada; ^2^ University of Waterloo, Waterloo, ON, Canada; ^3^ Schlegel‐UW Research Institute for Aging, Waterloo, ON, Canada

## Abstract

**Background:**

A Virtual Community of Practice (VCoP) can offer education and peer support for family and friend caregivers of people living with dementia. A community of practice consists of a group of people who share common challenges and want to enhance their knowledge through regular interactions. Namaste Care, a psychosocial multisensory program, is an example of an intervention well‐suited for delivery through a VCoP. Some program activities include massages, listening to music, telling stories, and having snacks and beverages. The purpose of this study is to explore the development of a VCoP, composed of an interactive website, a toolkit, and discussion forums, for family and friend caregivers of people with dementia in collaboration with people with lived experience, and its preliminary evaluation.

**Method:**

A qualitative descriptive design was used. A total of eight workshop sessions were held virtually. Research partners (i.e., nine caregivers and one person living with dementia) were located across Canada. Each research partner attended two workshop sessions by Zoom. Each workshop session included two to three research partners. In the evaluation phase, two caregivers completed an interview at the end of the intervention to explore their experiences with the VCoP. Thematic analysis was used to analyze data.

**Result:**

Research partners perceived the initial website provided helpful information related to strategies to meaningfully engage and stimulate people living with dementia. Research partners provided further recommendations including: (a) resources available in different languages; (b) additional tailoring of activities to different stages of dementia and cultural considerations; (c) videos on how to communicate and interact with persons living with dementia; and (d) self‐care resources for caregivers. Caregivers reported that they enjoyed being part of VCoP. The discussion forums were helpful to learn more about dementia and meet other caregivers in similar situations. They appreciated receiving resources which made it easy to use the adapted Namaste Care approach including the Namaste Care Toolbox with supplies, the training guide, and checklists.

**Conclusion:**

This project delivers a practical resource for caregivers, created in collaboration with people with lived experience, to support people living with dementia.

